# Expression of Caveolin 1 Is Enhanced by DNA Demethylation during Adipocyte Differentiation. Status of Insulin Signaling

**DOI:** 10.1371/journal.pone.0095100

**Published:** 2014-04-21

**Authors:** Sara Palacios-Ortega, Maider Varela-Guruceaga, Fermín Ignacio Milagro, José Alfredo Martínez, Carlos de Miguel

**Affiliations:** 1 Department of Biochemistry and Genetics, University of Navarra, Pamplona, Spain; 2 Department of Nutrition Food Science and Physiology, University of Navarra, Pamplona, Spain; 3 Physiopathology of Obesity and Nutrition CIBERobn, Carlos III Health Research Institute, Madrid, Spain; Virgen Macarena University Hospital, School of Medicine, University of Seville, Spain

## Abstract

Caveolin 1 (Cav-1) is an essential constituent of adipocyte caveolae which binds the beta subunit of the insulin receptor (IR) and is implicated in the regulation of insulin signaling. We have found that, during adipocyte differentiation of 3T3-L1 cells the promoter, exon 1 and first intron of the Cav-1 gene undergo a demethylation process that is accompanied by a strong induction of Cav-1 expression, indicating that epigenetic mechanisms must have a pivotal role in this differentiation process. Furthermore, IR, PKB-Akt and Glut-4 expression are also increased during the differentiation process suggesting a coordinated regulation with Cav-1. Activation of Cav-1 protein by phosphorylation arises during the differentiation process, yet in fully mature adipocytes insulin is no longer able to significantly increase Cav-1 phosphorylation. However, these long-term differentiated cells are still able to respond adequately to insulin, increasing IR and PKB-Akt phosphorylation and glucose uptake. The activation of Cav-1 during the adipocyte differentiation process could facilitate the maintenance of insulin sensitivity by these fully mature adipocytes isolated from additional external stimuli. However, under the influence of physiological conditions associated to obesity, such as chronic inflammation and hypoxia, insulin sensitivity would finally be compromised.

## Introduction

Knowledge of the regulatory mechanisms involved in adipocyte differentiation and hypertrophy is essential to understand the nexus between obesity and insulin resistance and will help to design new therapeutic approaches and targets.

There is growing evidence pointing to the multi-factorial origin of complex diseases like obesity and type 2 diabetes, with a particular focus on epigenetic modifications, which are considered the interface between genetics and the environment [1]–[2]. Recent publications have revealed the impact of different external factors on the epigenetic marks and their contribution to determine susceptibility to obesity and insulin resistance [3]. In this way, DNA methylation or histone tail modifications might be key elements for understanding obesity onset and development, considering their crucial role in gene expression and chromatin architecture. In fact, there are recent evidences of epigenetic involvement in the regulation of adipogenesis [4]–[5]. In particular, DNA methylation provides a stable tissue-specific gene regulatory system, capable of controlling transcription factor accessibility and recruitment of co-repressors or co-activators to chromatin.

Previous studies published by our group pointed to Caveolin 1 (Cav-1) as one of the genes upregulated in rat visceral white adipose tissue, in response to a high fat diet [6]. However, this effect was attenuated after a long feeding period with this diet, suggesting a possible compensatory mechanism and relating Cav-1 to insulin resistance onset [7]–[8]. Caveolins are the main structural and functional components of caveolae, flask shaped invaginations of the plasma membrane implicated in processes such as endocytosis, apoptosis, cholesterol homeostasis and cell signaling [9]. Cav-1 is an essential constituent of adipocyte caveolae and has been related to the compartmentalization and integration of several signal transduction pathways in these cavities [10]. This molecule is a 22 kDa integral membrane protein with a scaffolding domain that enhances the interaction between receptors and downstream signaling proteins, enabling a more efficient signal transduction. Indeed, there has been extensive evidence for Cav-1 implication in insulin signaling through direct binding to the beta subunit of the insulin receptor (IR) [11]. Cav-1 is activated by IR tyrosine kinase activity in response to insulin [12]., and stabilizes the receptor to maintain its activity in order to promote glucose uptake through Glut-4 translocation to caveolin-rich membrane fraction [13]. Furthermore, Cav-1 null mice show defective response to insulin in adipocytes, attributed to a 90% decrease in IR protein content [14], and dysregulation in lipid accumulation. These animals are resistant to diet-induced obesity, present associated white adipose tissue atrophy and exhibit reduced fat pads, hypertriglyceridemia, hyperinsulinemia and insulin resistance [15]. Previous studies have reported that, in some tumors, Cav-1 expression is regulated by the methylation of its promoter [16]–[17].

The purpose of the present study was to analyze epigenetic methylation changes in the Cav-1 gene during adipogenesis in relation to its expression and the connection of these changes with the insulin signaling pathway.

## Materials and Methods

### Cell Culture and Sample Extractions

3T3-L1 preadipocytes (ATCC, Rockville, MD) were seeded in 6-wells plates and grown in Dulbeco’s modified Eagle’s medium (DMEM, Invitrogen, Carlsbad, CL, USA) supplemented with 10% calf blood serum (CBS) and 1% penicillin/streptomycin in an incubator at 37°C, 5% CO_2_ and 95% humidity until reaching the second day post-confluence (d0). Adipocyte differentiation was then induced by incubating the cells with DMEM supplemented with 10% fetal bovine serum (FBS), insulin (1 µg/mL), dexamethasone (1 µM) and 3-isobutyl-1-methylxanthine (IBMX) (0.5 mM) for 48 hours. The medium was then replaced with DMEM containing 10% FBS and insulin (1 µg/mL) for an additional 48 hours. Cells were maintained in DMEM containing 10% FBS, until day 7 (d7) and 21 (d21), when adipocytes were harvested for further analyses. Total RNA and protein were extracted from each sample using DNA/RNA/Protein Mini Kit (Qiagen, Hilden, Germany) according to the manufacturer’s instructions.

### Oil Red O Staining

Intracellular lipid accumulation was assessed by Oil Red O Staining to estimate the degree of cell differentiation. 3T3-L1 preadipocytes (d0) and differentiated adipocytes at days 7 and 21 were washed twice with phosphate buffered saline (PBS) and fixed with formaldehyde 3, 7% during 2 hours. After washing three times with isopropanol 60%, cells were stained with Oil Red O (0.5% in isopropanol), diluted to 40% with water for 30 minutes at room temperature. Excess stain was removed by four washes with ethanol 70%. After drying, cells were photographed in a light microscope (Olympus Ck2, 40 X magnifications). Finally, incorporated Oil Red dye was released with isopropanol and spectrophotometrically quantified at 540 nm (Multiskan Spectrum, Thermo Electron Corporation, MA, USA).

### Cell Viability

Cell viability and proliferation was checked by MTT tetrazolium-based colorimetric assay. Briefly, 3T3-L1 preadipocytes (d0) and adipocytes (d7, d21) seeded in 96-well plates were washed twice with PBS and incubated for an hour with free serum DMEM containing 1 mg/mL of MTT in a humidified incubator at 37°C, 5% CO_2_. After medium removing, incorporated MTT was dissolved by adding DMSO (100 µl) and quantified spectrophotometrically at 540 mn (Multiskan Spectrum, Thermo Electron Corporation).

### Methylation Analysis of Cav-1 and IR Genes

The region of the Cav-1 gene studied encompasses from 619 bp 5′ to 1272 bp 3′ of the ATG codon (Figures S1 and S2), and includes two CpG islands located in the proximal promoter, exon 1 and the first intron. The region of the IR gene studied encompasses from 251 bp 5′ to 447 bp 3′ of the ATG codon (Figure S3), and includes 40 CpG sites located in the first exon and the first intron. Genomic DNA was extracted from preadipocytes (d0) and mature adipocytes (d21) and purified using the QIAamp DNA kit (Qiagen). Methylation of CpG dinucleotides was determined by MassArray Epityper technique (Sequenom Inc., San Diego, CA, USA) in the Central Research Unit of the School of Medicine (UCIM) of the University of Valencia (Spain). Measurements were performed in triplicate and data are presented as methylation percentages. From a total of 70 CpG dinucleotides present in the Cav-1 gene sequence analyzed, only 9 could not be analyzed.

### Real Time RT-PCR

Total RNA extracted from 3T3-L1 preadipocytes (d0) and adipocytes (d7, d21) was treated with DNase I (DNA-Free Kit, Ambion, Carlsbad, CA, USA) to remove possible contaminating genomic DNA before proceeding to reverse transcription. Two micrograms of purified total RNA were employed for first strand cDNA synthesis using M-MLV reverse transcriptase (Invitrogen) and random primers (Invitrogen), according to the manufacturer’s instructions. Specific TaqMan probes (Applied Biosystems, Foster City, CA, USA) were used to perform real-time PCR (ABI Prism 7300 HT Sequence Detection System, Applied Biosystems) for caveolin 1 (Cav-1,Mm_00483057_m1), insulin receptor (IR, Mm01211875_m1), Protein Kinase B (AKT-2, Mm02026778_g1) and glucose transporter type 4, (Glut-4, Mm00436615_m1). The cDNA was amplified in the following conditions: 50°C for 2 min and 95°C for 10 min, followed by 40 cycles of 15 s at 95°C and 1 min at 60°C, using the TaqMan Universal PCR Master Mix (Applied Biosystems). A probe for cyclophilin A (Ppia, Mm02342430_g1) was used as invariant internal control for RT-PCR efficiency and subsequent normalization. Relative gene quantification was calculated by the 2^−ΔΔCt^ method. Data were referred to day 0 as the calibrator sample.

### Western Blot Analysis

Proteins extracted from 3T3-L1 preadipocytes (d0) and adipocytes (d7, d21) were subjected to Western blot analysis performed with specific primary antibodies for Cav-1 (sc-894, Santa Cruz Biotechnology, Dallas, TX, USA), IR (sc-711, Santa Cruz Biotechnology), AKT (#9272, Cell Signaling, Beverly, MA, USA) and Glut-4 (G4048, Sigma Aldrich, St. Louis, MO, USA). Total proteins were separated by SDS-PAGE before transferring them to a nitrocellulose membrane (Hybond-C Extra, Amersham-Pharmacia, Amersham, UK). The membrane was then blocked with 5% milk in 0.05% Tris buffered saline with Tween 20 (TBST) for 2 hours and incubated overnight with the suitable primary antibody dilution at 4°C. Next, the membrane was washed three times with TBST to remove not bound antibodies after hybridizing it with horseradish peroxidase (HRP)-conjugated secondary antibody at the appropriate dilution. Finally, after three washing steps, specific immunoreactive bands were detected by a chemiluminescent ECL assay kit (ECL Western blotting, Amersham-Pharmacia). Specific secondary HRP-labeled rabbit (170-6515, Bio-Rad, Hercules, CA, USA) or mouse (NXA931, GE Healthcare, Little Chalfont, UK) antibodies were employed depending on the primary antibody origin. β-Actin (A1978, Sigma-Aldrich) was used as an invariant internal control for sample normalization.

The same methodology was followed to determine relative protein activation before and after stimulating cells with 50 nm insulin for 10 minutes. Specific primary anti-phospho protein antibodies for Cav-1 (Tyr14, 611339, BD Transduction Laboratories, Franklin Lakes, NJ, USA), IR (Tyr1146, #3021, Cell Signaling) and AKT (Ser473, #9271, Cell Signaling) were used.

### 2-[C^14^]-Deoxyglucose Uptake Assay in 3T3-L1 Preadipocytes and Adipocytes

2-[C^14^]-deoxyglucose uptake was analyzed in 3T3-L1 preadipocytes (d0) and differentiated adipocytes (d7, d21), at both basal state and after insulin stimulation. At first, cells were incubated in DMEM free of serum and glucose for 2 hours in a humidified incubator at 37°C, 5% CO_2_. Subsequently, cells were incubated in the presence or in the absence of insulin (50 µM) for 10 min. 2-C^14^.-deoxyglucose uptake was then initiated by the addition of DMEM free of serum and glucose containing 2-deoxyglucose (50 µM) and 2-C^14^.-deoxyglucose (0.075 µCi/mL, ARC0111, American radiolabeled, Saint Louis, MO, USA). Cells were incubated for 10 minutes at 37°C, 5% CO_2_ and the reaction was terminated by washing cells three times with pre-cold PBS containing 0.05 M glucose. Next, cells were incubated with a lysis buffer (0.1 M NaOH, 0.1% SDS) at 37°C for two hours and the cell lysate (100 µL) was transferred to a tube containing 2 mL of scintillation liquid for radioactivity counting using a scintillation counter (Wallac 1214 Rackbeta Counter, Perkin Elmer Life Sciences, Waltham, MA, USA). 2-Deoxyglucose uptake is reported as [C^14^] radioactivity, normalized to protein content from the remaining cell lysate, as determined by bicinchoninic acid (BCA) analysis (Pierce, Rockford, IL, USA). Measurements were performed in triplicate under conditions where hexose uptake was linear.

### Supernatant Adipokine Determination

Adiponectin (Cat. #EZMAKP-60K), Interleukin 6 (IL-6) (Cat. #EZMIL6) and Leptin (Cat. #EZML-82K) secreted to the culture medium from 3T3-L1 preadipocytes (d0) and adipocytes (d7, d21), were quantified by using ELISA kits from Millipore (Billerica, MA, USA), following the manufacturer’s instructions.

### Statistical Analysis

Data are expressed as mean ± standard error of the mean (S.E.M). Depending on the number of samples analyzed, data were compared using the ANOVA for a single factor and HSD-Tukey tests or applying the Wilcoxon signed rank test. All analyses were performed using SPSS version 15.0 for Windows (Chicago, IL, USA).

## Results

### Regulation of Adipokine Secretion during Adipogenesis

**Figure 1:**
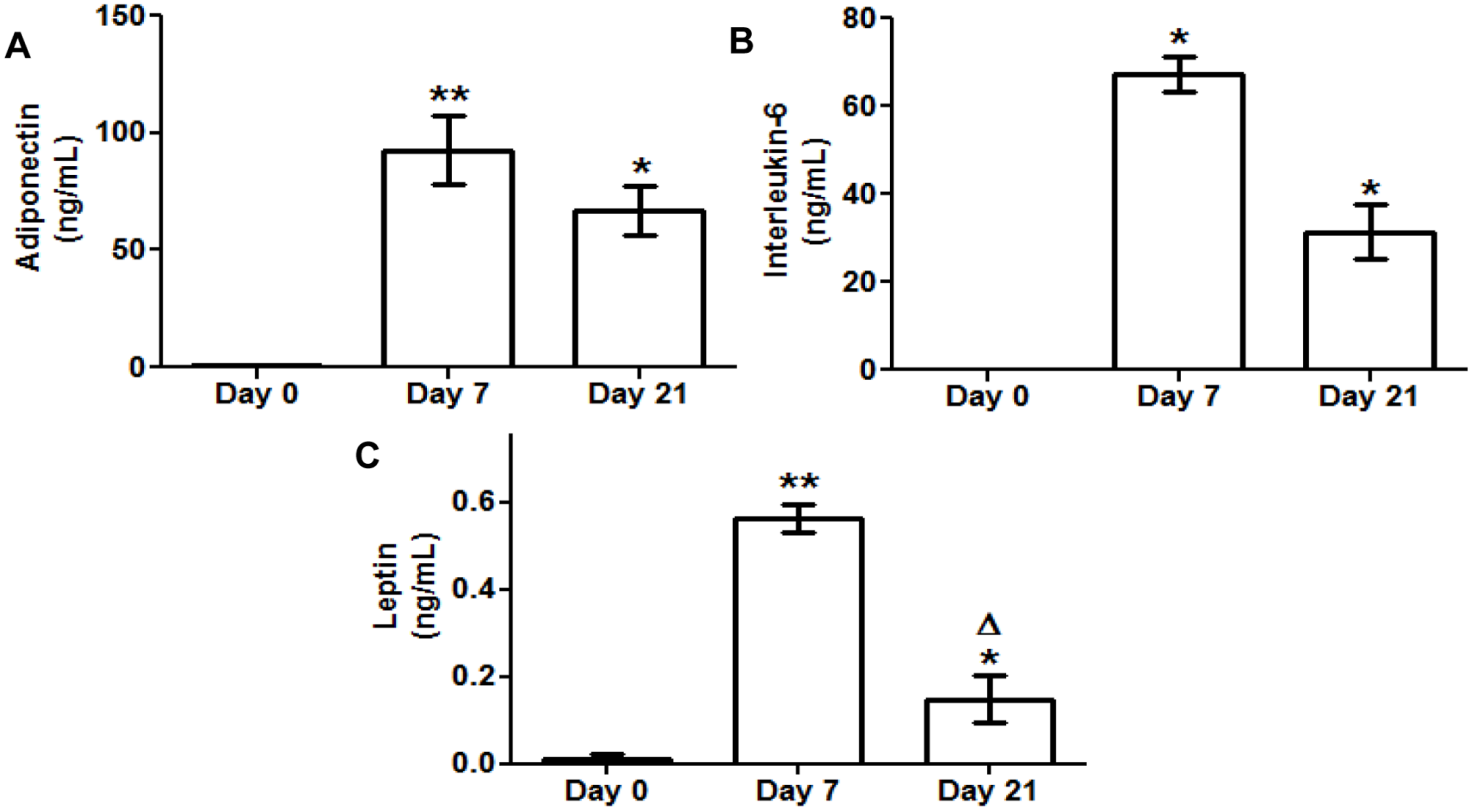
Secreted adiponectin interleukin 6 (IL-6) and leptin levels during 3T3-L1 cell adipogenesis. Culture medium samples of 3T3-L1 preadipocytes (d0) and adipocytes (d7, d21) were obtained for adipokine ELISA assay. Data are means ± SEM of the concentration (ng/mL) of each adipokine secreted to the cell medium. (A) Adiponectin (B) Interleukin 6 (IL-6) (C) Leptin. Groups were compared using the Wilcoxon signed rank test. Data are referred to day 0 *. p≤0.05, **. p≤0.01 or to day 7 Δ. p≤0.05.

Specific ELISA assays for adiponectin, IL-6 and leptin were carried out after harvesting the culture medium from 3T3-L1 preadipocytes (d0) and adipocytes (d7, d21). The secretion of all three adipokines was significantly increased during adipogenesis (d7, d21), reaching a maximum level at day 7 (Figure 1).

### Caveolin 1 Gene Undergoes Hypomethylation in Response to Adipogenesis

**Figure 2:**
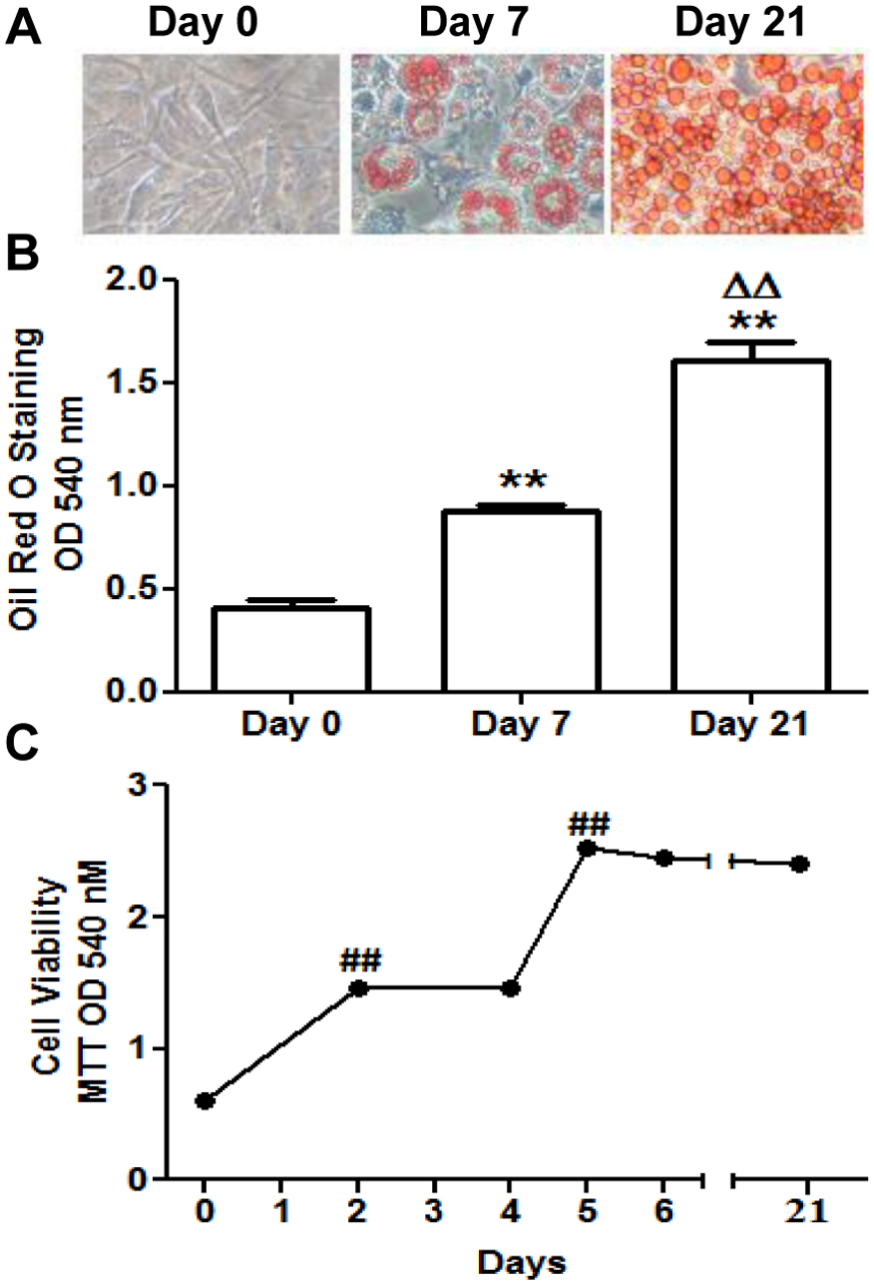
3T3-L1 cell differentiation and viability. (A) Cells were photographed at days 0, 7 and 21 of their adipocytic differentiation after Oil Red O staining using a light microscope (40X magnification). Data are means ± SEM of dye OD at 540 nm. (B) Intracellular triglycerides quantification at days 0, 7 and 21 using Oil Red O Staining. (C) Cell viability measurement at days 0, 2, 4, 5, 6 and 21 using MTT assay. Data are means ± SEM of dye OD at 540 nm. Groups were compared using the ANOVA for a single factor and HSD-Tukey tests. Data from (B) are referred to day 0 **. p≤0.01 and to day 7 ΔΔ. p≤0.01. Data from (C) are referred to the previous checking point ##. p≤0.01.

3T3-L1 preadipocytes differentiated to adipocytes in response to the established procedure. Lipid accumulation was verified by Oil-red O staining (Figure 2 A, B). A double clonal expansion was observed following the start of 3T3-L1 differentiation and cell viability was not altered along this process (Figure 2C).

**Figure 3:**
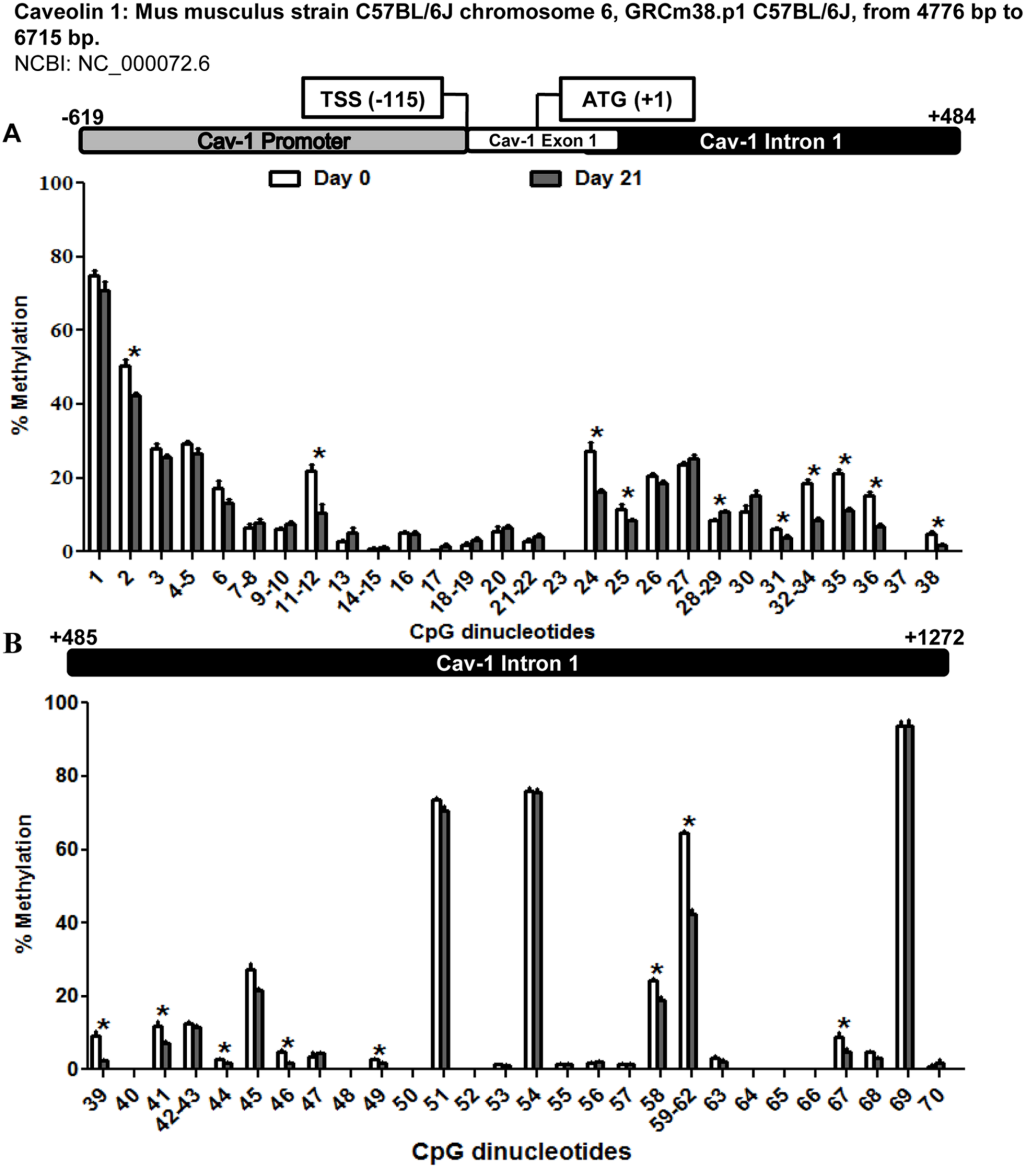
Methylation levels of the CpG dinucleotides in the caveolin 1 promoter exon 1 and intron 1 throughout 3T3-L1 cell adipogenesis. The methylation level of 70-L1 adipocytic differentiation. MassARRAY system was used for the quantitative methylation analysis. (A) CpG 1 to 38 (B) Cpg 39 to 70 from the sequence under study. Data are means ± SEM of the methylation percentage of each CpG dinucleotide specified in the figure at days 0 and 21 of 3T3-L1 adipocyte differentiation. Groups were compared using the Mann-Whitney U test. Significant differences between day 0 and day 21 *. p≤0. 05. Gene structure is schematized over the graphs indicating the transcription start site (TSS) and the initiation codon (ATG) position.

This experimental model was used to assess possible changes in the methylation pattern of the promoter, exon 1 and intron 1 of Cav-1 (–619 bp to +1272 bp from initiation codon ATG) during adipogenesis. As shown in figure 3, the region under study displayed an overall loss of methylation from preadipocytes (d0) to mature adipocytes (d21), being especially significant from position +27 to +600 pb of the sequence, corresponding to the first intron of the gene, and encompassing CpG 24 to 46.

The same experimental design was performed to analyze the methylation pattern of the first exon and first intron of IR (–251 bp to +447 bp from initiation codon ATG) during adipogenesis. Five of the forty two CpG studied showed significant differences, with four of them, located in the first exon, displaying demethylation in mature adipocytes in comparison with preadipocytes (Figure S4).

### Caveolin 1 and Insulin Signaling Pathway Intermediates Expression Levels Increased during Adipogenesis

**Figure 4:**
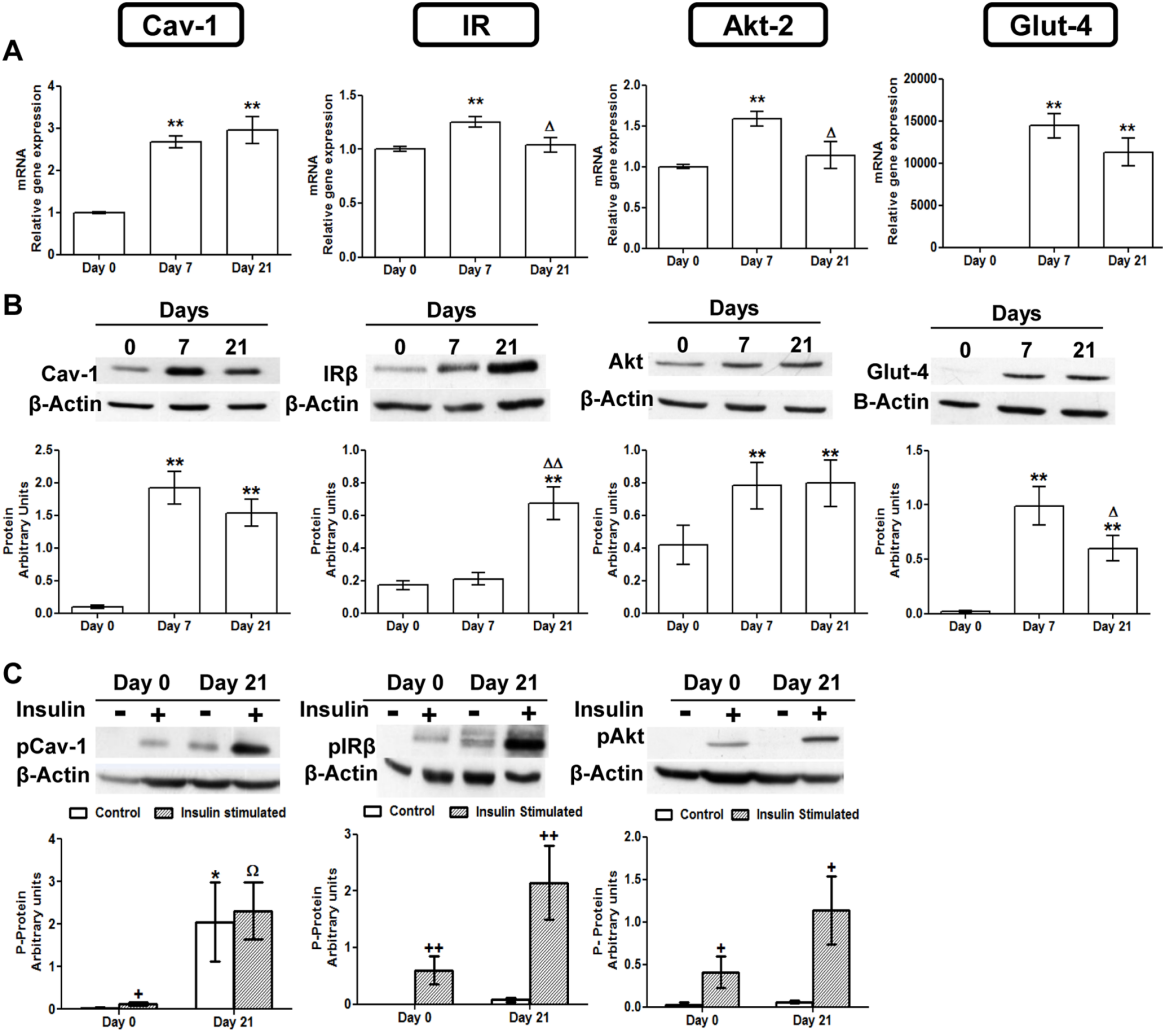
Caveolin 1 and insulin signaling intermediaries expression and activation throughout 3T3-L1 cell adipogenesis. (**A**) mRNA levels. Data are means ± SEM of the ratio between each gene and cyclophilin expression at differentiation days 0, 7 and 21. Groups were compared using the ANOVA for a single factor and HSD-Tukey tests. (**B**) Protein levels. Data are means ± SEM of the ratio between each protein and β-Actin expression at differentiation days 0, 7 and 21. Groups were compared using the Wilcoxon signed rank test. (**C**) Cav-1 IR and AKT phosphorylation levels. Results represent data from cells before and after stimulation with insulin (50 nM, 10 minutes). Data are means ± SEM of the ratio between pCav-1, pIRβ and pAKT protein and β-Actin expression in the differentiation days 0 and 21. Groups were compared using the Wilcoxon signed rank test. Data are referred to day 0 *. p≤0.05, **. p≤0.01 or to day 7 Δ. p≤0.05, ΔΔ. p≤0.01. Data from insulin stimulated groups (C) were compared to their unstimulated control group +. p≤0.005 ++. p≤0.01 or to the insulin stimulated undifferentiated group Ω. p≤0.05.

The expression profiles of Cav-1 and three insulin signaling pathway intermediates (IR, AKT-2 and Glut-4) were screened by real-time PCR during adipogenesis. The checking points were established at days 0, 7 and 21 after the onset of the adipogenic stimulation. Interestingly, Cav-1 mRNA levels were significantly increased throughout adipocyte differentiation (d7, d21). IR, AKT-2 and Glut-4 exhibited a significant upregulation at day 7, although at day 21 only Glut-4 remained high (Figure 4A).

Western blot analyses for Cav-1, IR, AKT-2 and Glut-4 were also performed during the differentiation process. Similarly to mRNA, Cav-1 protein was significantly increased in mature adipocytes (d7, d21) in comparison to preadipocytes (d0). Furthermore, AKT-2 and Glut-4 protein levels showed a similar pattern, whereas IR reached a significant increment only at day 21 (Figure 4B).

### Caveolin 1 and Insulin Signaling Pathway Intermediaries Activation is Increased in Mature Adipocytes

**Figure 5:**
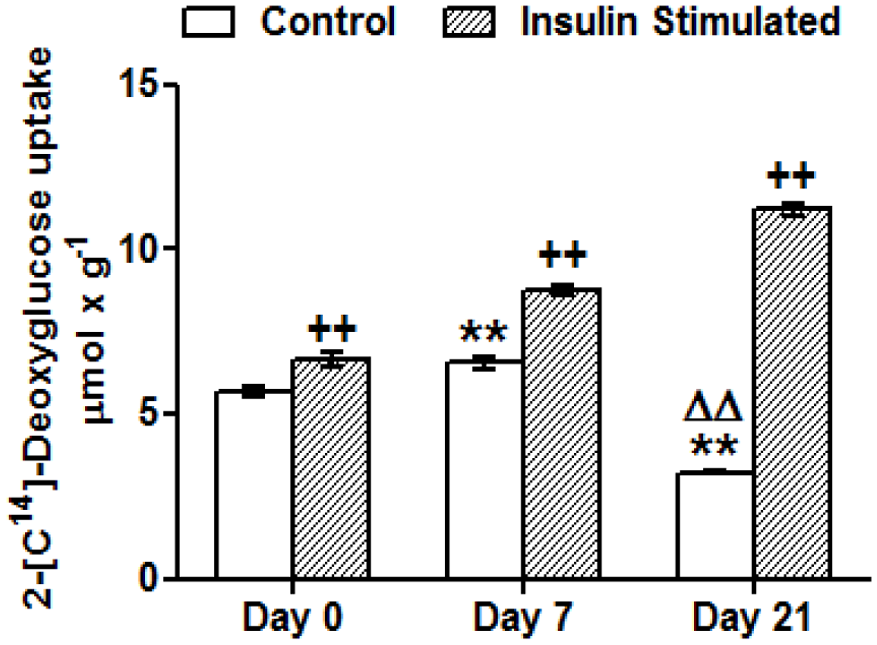
Deoxyglucose uptake throughout 3T3-L1 cell adipogenesis. Results represent deoxyglucose uptake by 3T3-L1 cells before and after insulin stimulation (50 nM, 10 min). Data are means ± SEM of 2-[C14]-deoxyglucose (in µmol) incorporated by cells after 10 minutes, adjusted by total protein in grams. Groups were compared using the ANOVA for a single factor and HSD-Tukey tests. Data are referred to control day 0 **. p≤0.01 or to control day 7 ΔΔ. p≤0.01. Data from insulin stimulated groups were compared to their unstimulated control group ++.p≤0 01.

Activation of Cav-1, IR and AKT-2 was measured by Western blot analysis using specific anti-phospho protein antibodies before and after insulin stimulation. As shown in figure 4C, basal level of Cav-1 phosphorylation was significantly increased in mature adipocytes (d21), whereas no basal activation was found in preadipocytes (d0). On the other hand, insulin response was positive in both stages, although the extent of activation was not significant at day 21. However basal phosphorylation of IR and AKT-2 was almost absent in preadipocytes and did not increased significantly at day 21. Similarly to Cav-1, both proteins increased the activation levels after insulin stimulation, and mature adipocytes (d21) also showed a higher response than undifferentiated cells (d0). Glut-4 activation was assayed by measuring glucose transport before and after insulin stimulation (Figure 5). Basal 2-deoxyglucose uptake was higher at day 7 whereas in fully mature adipocytes decreased. Insulin was able to promote hexose uptake either at basal stage (d0) or in differentiated cells (d7, d21). The fact that basal glucose uptake at day 21 was diminished made insulin effect more prominent.

## Discussion

The adipogenic process of the 3T3-L1 cells was accompanied by an increase in the secretion of three important adipokines: adiponectin, interleukin 6 (IL-6) and leptin and by a significant intralipid storage (Figure 1 and 2). Adiponectin is a hallmark of healthy mature adipocytes that contributes to insulin sensitivity and anti-inflammatory protection [18]. IL-6, an important inflammation mediator, has also been shown, in a non-pathological condition, to enhance PPARγ expression [19], favouring adipocyte differentiation. Leptin helps these cells to regulate adipocyte metabolic homeostasis, preventing excessive fat storage [20]. Nevertheless, it is important to consider that in our model, adipocytes differentiate isolated from the interaction with other cell types present in the adipose tissue, such as infiltrating immune cells, which can have a profound influence in the process. This factor can be critical in pathological situations like the chronic inflammation associated to obesity. In fact, there seem to be surfaceome differences between primary adipocytes from mouse models of obesity and 3T3-L1 cells [21], which would explain the variations in their response to external stimuli.

During adipogenic differentiation of the 3T3-L1 cells, we have found a significant induction of the Cav-1 gene accompanied by increased levels of Cav-1 protein (Figure 4A and B). The high expression of Cav-1 in mature adipocytes is something already well established [22] Indeed, previous works from our group had also shown an increase of Cav-1 mRNA and protein expression in whole white adipose tissue [6]–[7], and in isolated adipocytes [8] of rats with a diet-induced model of obesity. The general decrease in methylation observed in this study in the analyzed region of Cav-1 gene, points to an epigenetic regulation mechanism for this induction.

The region under study extends about 2 kb and encompasses 70 CpG sites distributed along the proximal promoter, the first exon and a substantial portion of the first intron (Figure 3). Particularly, the most significant differences affect the intronic fragment, where 23 of the total of 25 CpG sites that showed significant methylation losses are located (Figures S1 and S3). Sequence analysis revealed putative transcription regulatory elements near the significantly demethylated CpGs (Figure S2). Of special interest are those elements described as binding sites for factors active in the adipogenic process. One of the most notable is C/EBPβ (CpG 46) (Enhancer Binding Protein β), which is essential for the expression of pro-adipogenic genes such as C/EBPα and PPARγ (Peroxisome Proliferator Activated Receptor γ) [23]. C/EBPβ is also necessary as a pioneering factor for PPARγ binding to adipogenic hotspots [24]. The Cav-1 sequence also shows several possible recognition sites for the adipocyte-specific fatty acid binding protein AP2 (CpG 11–12, 49 and 59–62), which is necessary for the generation and maintenance of the adipogenic phenotype [25]. Sterol Regulatory Element Binding Proteins or SREBP (CpG 24) are E-box binding transcription factors involved in the regulation of lipogenesis and lipid homeostasis [26]. In fact, a role for SREBP has been already demonstrated in the transcriptional regulation of human Cav-1 [27]. In this context, it is especially relevant that Estrogen Receptor α or ERα (CpG 58), a nuclear protein implicated in glucose homeostasis, has been reported to silence Cav-1 gene through CpG hypermethylation of its promoter in human neuroblastoma cells [28]. Moreover, a recent study in breast cancer cell lines also demonstrated the direct relation between the expression of Cav-1 gene and the methylation level of certain CpG-rich regions [29]. This same region (CpG 58) also displays a recognition site for the Liver X receptor (LXRα) and the retinoic X receptor (RXRα), major transcriptional regulators of cholesterol, lipid and glucose metabolism in adipocytes, hepatocytes and macrophages [30]–[31], that have been described as insulin-sensitizing factors when acting as dimers [32] However, in human adipocytes from overweight individuals, Pettersson et al. [33] have recently suggested that LXR downregulates the expression of Cav-1 and other genes involved in insulin signalling, reducing glucose uptake in response to this hormone. Finally, it is interesting to underline the presence of two possible binding sites for hypoxia-inducible factor 1 (HIF-1) close to CpG sites that change their methylation status during adipogenesis (CpG 32–34, 49), since this factor has been related to adipocyte dysfunction under the hypoxic conditions associated to obesity [34].

In summary, there is a correlation between the significant reduction in the methylation level of Cav-1 gene during the adipogenic process and the accompanying increase in its expression, indicating that epigenetic changes may be an important regulatory mechanism in this differentiation process. In fact, DNA methylation, has been previously reported to regulate Cav-1 expression in breast cancer and T cell leukemia cell lines [16]–[17].

Since Cav-1 can be considered an adipocyte differentiation marker, and given that some of the hypomethylated sites lay on consensus sequences for key adipogenic transcription factors, it is conceivable that this epigenetic mechanism could be involved in the control of other adipogenic genes whose transcription can be regulated also by promoter methylation, such as PPARγ 35., FASN [36], or insulin [37]. Here we have used the same samples, after 21 days of adipocyte differentiation, to analyse the changes in CpG methylation in a 780 bp region overlapping the first exon and intron of the IR gene. In this case, we have found that only 5 out of 42 CpGs, showed a significant decrease in methylation (Figure S4), but the IR gene expression was not significantly induced at that stage. It will be interesting to study the methylation state of this sequence after 7 days of differentiation, when an increased expression of IR was detected (Figure 4A). However, although other authors have described some changes in the IR promoter methylation [38], to date there are no strong evidences of an association with its expression level, and the main regulatory mechanisms for the IR transcript seem to be post-transcriptional [39].

The involvement of Cav-1 in the regulation of insulin signal transduction is extensively documented. Multiple studies have demonstrated that IR colocalizes with Cav-1 in caveolae, and also that other downstream molecules such as IRS-1, Shc, GRB-2, SOS, Syp and PI3-kinase are recruited to these structures after insulin stimulation, enhancing the subsequent phosphorylation cascade [40]–[42].

Taking all these precedents into account, we have also measured Cav-1 activation and evaluated the state of the insulin signalling pathway. For this purpose we have analysed the expression and the activation of IR, PKB-Akt (protein kinase B) and Glut-4 (glucose transporter type 4) as an initial, middle, and final intermediary respectively. Our results show a significant induction of IR, PKB-Akt and Glut-4 expression that positively correlated with that of Cav-1, as part of the adipocyte differentiation process (Figure 4A). However, after 21 days of differentiation, although protein expression remained high (Figure 4B), only Cav-1 and Glut-4 gene induction were maintained, indicating a higher turnover for these proteins.

It is interesting the fact that during adipogenesis Cav-1 became significantly phosphorylated and therefore activated, while on the contrary, neither IR nor PKB-Akt phosphorylation changed (Figure 4C). This would explain why insulin, which promotes Cav-1 phosphorylation in the immature preadipocytes, has little effect after 21 days of differentiation (Figure 4C). The reduced insulin stimulation of Cav-1 in mature adipocytes has been attributed to the fraction of this protein directly associated with IR [43]. Therefore, the basal activation of Cav-1 during adipogenesis might be consequence of alternative regulatory mechanisms that could represent a way to prepare cells for a prompt and more efficient response to insulin and other pro-adipogenic signals. On the other hand, IR and PKB-Akt activation by insulin, which is patent in the immature cells, becomes highly increased after adipocyte maturation (Figure 4C).

Cav-1 also has been previously related to Glut-4 function by participating in the translocation of this glucose transporter back and forth between internal vesicles and the plasma membrane [44]. Insulin stimulation of the adipocytes increases the amount of Glut-4 protein in the caveolin-rich membrane fractions, whereas Cav-1 depletion reduces the quantity of Glut-4 protein in the 3T3-L1 cells, enhancing its degradation and eventually affecting the insulin response [45]. As expected, and in correlation with the increased expression of Glut-4, we were able to detect a greater insulin-stimulated glucose uptake in the differentiated cells compared to the preadipocytes (Figure 5). However, we have also observed how the basal glucose uptake was increased after 7 days of differentiation and, in accordance with previous reports [46], it became notably reduced in more mature cells after 21 days (Figure 5). This insulin-independent glucose transport has been mainly attributed to the facilitative glucose transporter 1 (Glut-1), since the adipocyte specific isoform Glut-4 remains mainly into intracellular compartments in unstimulated cells. In preadipocytes, Glut-4 is virtually absent and Glut-1 is almost entirely located in the plasma membrane. The adipocyte differentiation process induces the synthesis of Glut-4, which initially could be responsible for the increased basal glucose uptake (Figure 5, day 7). Nevertheless, the differentiation process ends with the internalization of Glut-1 and the formation of a large intracellular pool containing Glut-1 and the adipocyte-specific insulin-sensitive Glut-4. This finding would explain the lower capacity of fully differentiated cells to internalize glucose under basal conditions, and the higher glucose uptake observed when insulin stimulation triggers the rapid mobilization of both transporters to the plasma membrane (Figure 5, day 21) [47].

Some studies have suggested that large fat cells are less responsive to insulin [48], pointing to mature hypertrophic adipocytes as one of the causes of insulin resistance [49]. However, we have seen here that in terms of signal activation, 3T3-L1 mature fully-differentiated fat cells maintain an adequate insulin response capacity after 21 days of differentiation, indicating that this long-term differentiation process does not contribute to the onset of adipocyte insulin resistance by itself. The simultaneous activation of Cav-1 that we report during this process could facilitate the maintenance of insulin sensitivity by the mature cells. Nonetheless, as pointed out before, the decisive influence of the cellular environment for the regulation of cellular functions, in an in vivo situation, such as inflammation or hypoxia, might explain these differences.

In summary, we have shown how the induction of Cav-1 gene during adipogenesis could be explained by the hypomethylation of sequences that can regulate transcription and how this is accompanied by an increase in Cav-1 activation. A parallel increase in the expression of several insulin signalling intermediaries, such as IR, PKB-Akt and Glut-4, suggests that similar epigenetic mechanisms could be involved in an orchestrated regulation. The activation of Cav-1 might contribute to the efficient insulin-response elicited by fully mature adipocytes after a long differentiation period.

## Supporting Information

Figure S1 **Genomic localization and nucleotide sequence of the Caveolin 1 gene analyzed in this work by MassArray Epityper technique.** CpG dinucleotides located in the proximal promoter, exon 1 and intron 1 of the Cav-1 gene (from −619 to 1272 pb from ATG codon (+1)) are shadowed. ATG codon indicates the Translation Start Site (CDS) and TSS indicates the Transcription Start Site. Due to the limitation of the technique, DNA methylation of some CpGs sites could not be measured (crossed out), or were measured together with others (indicated as boxed CpGs sites).
(PDF)

Figure S2 **Caveolin-1 CpG sites sequence localization, methylation changes during adipogenesis and possible regulatory transcription factors associated to these sequences** *.p≤0,05, **p≤0,01.
(PDF)

Figure S3 **Genomic localization and nucleotide sequence of the Insulin Receptor gene analyzed in this work by MassArray Epityper technique.** CpG dinucleotides are located in the exon and intron 1 of the Cav-1 gene (from −249 to 445 pb from ATG codon (+1)) are shadowed. ATG codon indicates the Translation Start Site (CDS). Due to the limitation of the technique, DNA methylation of some CpGs sites could not be measured (crossed out), or were measured together with others (indicated as boxed CpGs sites).
(PDF)

Figure S4 **Methylation levels of the CpG dinucleotides in the insulin receptor (IR) exon and intron 1 throughout 3T3-L1 cell adipogenesis.** The methylation level of 42 CpG sites in IR exon 1 and intron 1 were compared before and after 3T3-L1 adipocytic differentiation. MassARRAY system was used for the quantitative methylation analysis. Data are means ± SEM of the methylation percentage of each CpG dinucleotide specified in the figure, at days 0 and 21 of 3T3-L1 adipocyte differentiation. Groups were compared using the Mann-Whitney U test. Significant differences between day 0 and day 21 *. p≤0,05. Gene structure is schematized over the graphs, indicating the initiation codon (ATG) position.
(PDF)
