# Supplementary material for: Expression of Caveolin 1 Is Enhanced by DNA Demethylation during Adipocyte Differentiation. Status of Insulin Signaling
Source: PLoS One. 2014 Apr 21;9(4):e95100. doi: 10.1371/journal.pone.0095100 (PMC3994010; doi:10.1371/journal.pone.0095100)
Supplement: Figure S1 — Genomic localization and nucleotide sequence of the Caveolin 1 gene analyzed in this work by MassArray Epityper technique. CpG dinucleotides located in the proximal promoter, exon 1 and intron 1 of the Cav-1 gene (from −619 to 1272 pb from ATG codon (+1)) are shadowed. ATG codon indicates the Translation Start Site (CDS) and TSS indicates the Transcription Start Site. Due to the limitation of the technique, DNA methylation of some CpGs sites could not be measured (crossed out), or were measured together with others (indicated as boxed CpGs sites). (PDF) [file pone.0095100.s001.pdf]

**Caveolin 1: Mus musculus strain C57BL/6J chromosome 6, GRCm38.p1 C57BL/6J, from 4776 bp to 6715 bp.**

NCBI: NC_000072.6

```
ttgaacctct aagtaaaatt ttctgcagtt gaaaatactc aaagtgctta tggttactgt -661
                                              1                 2
ataacctgct ggtagggatt tcattttctt tattgcagag gcgcccacac agattgcttc -601
                                                               3
gttgtgttta tatttaaaaa aaaaaaaaat gttccctcag gttcccagcc atctcgcttc -541
                                              4     5
tatatctttc tctgtgaaac aaggagacag atcagttcta cgtgggcgaa ttgggagggg -481
                                           6
aggcagctta ggacagggca gaattctttc tgcagagccg gatgcccata ctgggcatct -421
                                                               7-8
ctgcagactc ttgggctccc tccacccctg ctgagatgat gcactgggaa aacacgcgct -361
                                                  9-10
ctcccctgga tgcctctctg taggtttata gctgggaaaa cgttgcctcg agtctaaaat -301
                                                                11-12
atctgcccaa actgcctagc tctgatgaag gctttctcac aggctctcag ctccccgccg -241
      13                                           14-15       16
gcactccccg ccctctgctg ccagaacctt ggggatgtgc ctagacccgg cgcagagcac -181
                                       17
gtcctagcca actgagagca gaacaaaccc ctggcgaaca gccaagaggc tccctcccag -121
      18-19       20
ccaccgcccc ccgccagcgc ctttcccccc tctatacaat acaagatctt ccttcctcag -61
    TSS                                                     21-22
ttctcttaaa tcacagccca gggaaacctc ctcagagcct gcagccagcc acgcgccagc -1
CDS               23      24
ATGtctgggg gcaaatacgt agactccgag gtaggcttgg gtgagggctc caaagtgcct +60
                               25             26          27
gcttttgcta tcttccactc caaatgctcg gactgctttc ctcgccccag cccgctctcc +120
     28-29      30
accttcgcgg cactcgagct ggcaccttgg aggaatgggc tgggtgggga ggtgaaaagc +180

taggaatgtc ttagggcttc cttttggaga ggagaccaat ggagccaaaa ggggaaagaa +240
      31                                         32-33-34
ggagacccgg tctgccagac ttagaaaagt tttccagatg acacgttcgc gagaccccac +300
  35        36
tgcggtccag ccccgtaccta ctgggaggct tagggctgct gctcagtgtg tgtgtgtgtg +360
                                                      37
tgtgtgtgtg tgtgtgtgtg tgtgtgtgtg tgtgtgtgtg tgtgttcggg ggaaggagat +420
```

```
tcatttggac agtgtccccc caccccaacc ccccaggagc ctaaagtggc tggcttgggc +480

  38     39                 40        41
agcggcatgc gtgtattgca cttagcgaag ttctcgagaa cttcaatagt tttttctgcc +520
              42-43          44
cctgagaggc ccggcgggg ggagttccga ggtgggggaa gcctccttcc tccctcacaa +600
       45                                        46         47
gtaccaccgc cattgtcaac tccttcttgg cactcaggtg gactaaacgt ggagacccg +660
                  48
gttcccaaat agggctacga ttcagcttgg agtagacagc ctgagagtga tctaagtaag +720

ggtgggtcag gcagttcttc agatggcatg tcctctgggg catgggggtg ggggtgacag +780

gtcttggcac atgcctcagc ctaaagaagt atgttagatt gtgagccact tattcacccc +840
  49
cacggcctaa gaaaaaccag ggagtaaagg tgggttttaa aagggggtag gggctgtggc +900
                  50                                51
aggtgcaagg aagaagcacg gagggacagt gaagatggga ccattacgtc tgtcagtttg +960

gtccagagtg atcttgataa tgttgaaagg gaccttagga cagtgttaag ggttaagaga +1020
       52                                   53
gggaccacgg agcaggtgag gttgtggggt cataggtctg gcggagagaa ggaatacaga +1080
            54                                  55         56
ggaggaggaa cgggaacccc agcccctggg ctggggagtc agggccgaga gccgtgggga +1140
         57          58              59-60-61-62           63
gcaggagggc gcatgggact tcgccctgac ccctggcggc gggcgcggga ggcaggcgct +1200
              64                 65  66                      67
ctctgcctgg taccgagggg tgtggtgtcc tccgctccgt cctcttaaaa agcttgcagc +1260
                                68                          69
gctggagttt tctgtgtgca agcctttcct tcgaggttga agctagcaaa agttgtagcg +1320
             70
ccaggctacc cccgcacagc caggctgact cttgactctt gtttttcttt ctgaactttt +1380
```

**Figure S1: Genomic localization and nucleotide sequence of the Caveolin 1 gene analyzed in this work by MassArray Epityper technique.** CpG dinucleotides located in the proximal promoter, exon 1 and intron 1 of the Cav-1 gene (from -619 to 1272 pb from ATG codon (+1)) are shadowed. ATG codon indicates the Translation Start Site (CDS) and TSS indicates the Transcription Start Site. Due to the limitation of the technique, DNA methylation of some CpGs sites could not be measured (crossed out), or were measured together with others (indicated as boxed CpGs sites).
