# Supplementary material for: Expression of Caveolin 1 Is Enhanced by DNA Demethylation during Adipocyte Differentiation. Status of Insulin Signaling
Source: PLoS One. 2014 Apr 21;9(4):e95100. doi: 10.1371/journal.pone.0095100 (PMC3994010; doi:10.1371/journal.pone.0095100)
Supplement: Figure S2 — Caveolin-1 CpG sites sequence localization, methylation changes during adipogenesis and possible regulatory transcription factors associated to these sequences *.p≤0,05, **p≤0,01. (PDF) [file pone.0095100.s002.pdf]

Figure S2: Caveolin-1 CpG sites sequence localization, methylation changes during adipogenesis and possible regulatory transcription factors associated to these sequences *.p≤0,05, **p≤0,01.

	Seq. Location	Day 0(%Met)	Day 21(%Met)	Change(% Met)	p	Trascription factors
**CpG 1**	-618	72,58	70,93	1,65	0,73	
**CpG 2**	-601	47,92	42,20	5,72	0,19	
**CpG 3**	-546	27,92	25,53	2,38	0,19	
**CpG 4-5**	-500, -494	29,25	26,53	2,72	0,11	
**CpG 6**	-442	17,33	13,13	4,20	0,19	
**CpG 7-8**	-366, -364	6,50	7,73	-1,23	0,56	
**CpG 9-10**	-320, -312	6,08	7,37	-1,28	0,19	
**CpG 11-12**	-245, -242	21,83	10,53	11,30	*0,016	CBF, AP-2, WT1
**CpG 13**	-232	2,83	5,20	-2,37	0,19	
**CpG 14-15**	-193, -190	0,60	0,93	-0,33	0,42	
**CpG 16**	-181	5,13	4,70	0,43	0,69	
**CpG 17**	-146	0,33	1,40	-1,07	0,31	
**CpG 18-19**	-116, -108	1,67	2,92	-1,25	0,34	
**CpG 20**	-103	5,50	6,33	-0,83	0,69	
**CpG 21-22**	-8, -6	2,87	4,20	-1,33	0,15	
**CpG 23**	+18					
**CpG 24**	+27	27,25	16,13	11,13	*0,029	TFII-I, SREBP
**CpG 25**	+89	11,53	8,42	3,12	*0,016	p300
**CpG 26**	+103	20,47	18,60	1,87	0,15	
**CpG 27**	+113	23,46	25,07	-1,61	0,29	
**CpG 28-29**	+126, +128	8,53	10,73	-2,20	**0,008	WT1, CBF, HES1,deltaEF1
**CpG 30**	+135	10,67	15,25	-4,58	0,20	
**CpG 31**	+248	6,08	5,40	0,68	0,19	
**CpG 32-34**	+284, +288, +291	18,50	8,50	10,00	*0,016	TGIF, AhR, Arnt, HIF-1, AP1, CBF
**CpG 35**	+303	21,08	11,12	9,97	*0,016	CBF
**CpG 36**	+313	15,17	6,78	8,38	*0,016	
**CpG 37**	+407					
**CpG 38**	+483	4,83	1,67	3,17	*0,016	AhR, Arnt, CBF.
**CpG 39**	+490	9,33	2,33	7,00	*0,016	AhR, Arnt, HIF-1, USF2
**CpG 40**	+506					
**CpG 41**	+515	11,92	6,67	5,25	*0,016	p300
**CpG 42-43**	+552, -556	12,42	13,80	-1,38	0,91	
**CpG 44**	+568	2,88	1,80	1,08	*0,016	CBF, YY1, WT1
**CpG 45**	+608	29,33	21,47	7,87	*0,032	CBF, HIF-1, AhR, Arnt
**CpG 46**	+648	4,83	1,67	3,17	*0,016	C/EBP beta, c-ETS-1
**CpG 47**	+659	3,42	4,47	-1,05	0,73	

**CpG 48**	+678					
**CpG 49**	+843	2,88	1,80	1,08	*0,016	CBF, AhR, Arnt, HIF-1, AP-2
**CpG 50**	+919					
**CpG 51**	+947	71,71	70,70	1,01	0,41	
**CpG 52**	+1028					
**CpG 53**	+1062	1,42	1,20	0,22	0,41	
**CpG 54**	+1091	75,88	75,67	0,21	1,00	
**CpG 55**	+1126	1,33	1,43	-0,10	0,73	
**CpG 56**	+1133	1,63	2,03	-0,41	0,56	
**CpG 57**	+1150	1,33	1,43	-0,10	0,73	
**CpG 58**	+1162	24,42	19,03	5,38	*0,016	ER, LXR alpha, RXR alpha
**CpG 59-62**	+1177, +1180, +1184, +1186	64,42	42,30	22,12	*0,016	AP-2, AP-1, CBF
**CpG 63**	+1197	3,25	2,10	1,15	0,29	
**CpG 64**	+1214					
**CpG 65**	+1233					
**CpG 66**	+1238					
**CpG 67**	+1260	8,79	4,87	3,93	*0,032	p300
**CpG 68**	+1292	4,75	3,07	1,68	0,06	
**CpG 69**	+1319	93,79	92,57	1,22	0,73	
**CpG 70**	+1333	0,92	1,93	-1,02	0,41	
