# Supplementary material for: Expression of Caveolin 1 Is Enhanced by DNA Demethylation during Adipocyte Differentiation. Status of Insulin Signaling
Source: PLoS One. 2014 Apr 21;9(4):e95100. doi: 10.1371/journal.pone.0095100 (PMC3994010; doi:10.1371/journal.pone.0095100)
Supplement: Figure S3 — Genomic localization and nucleotide sequence of the Insulin Receptor gene analyzed in this work by MassArray Epityper technique. CpG dinucleotides are located in the exon and intron 1 of the Cav-1 gene (from −249 to 445 pb from ATG codon (+1)) are shadowed. ATG codon indicates the Translation Start Site (CDS). Due to the limitation of the technique, DNA methylation of some CpGs sites could not be measured (crossed out), or were measured together with others (indicated as boxed CpGs sites). (PDF) [file pone.0095100.s003.pdf]

Insulin Receptor: Mus musculus strain C57BL/6J chromosome 8, GRCm38.p1 C57BL/6J, from 19521 bp to 20367 bp.

NCBI Reference Sequence: NC_000074.6

```
                                                     1       2         3
ggacaggaga cgcaccgcgg tgagctctgg actctgggat tgcgggcacg ggaccgggcc -232
              4-5-6            7-8-9                          10
tgggtgacct gcgggccgcg ccacggtgtt gcttgcggcc gaggcctctg tgctcttccc -172
                                            11            12-13
gggactgtcc ccagggccct ctaggctgga gagctgcggc ctgtgagcca cgggcgtgga -112
            14-15-16      17                     18
agagaaggac gtgcggcccc gagcgcctct ccagagacct tctcacggag tatgtcccca -52
          19-20-21          22-23                          CDS
gtaggccggc gtggcgtgct ctgatcgccg gggtcccagc actcctactg ctATGggctt +8
24                    25  26                   27-28-29
cgggagagga tgtgagacga cggctgtgcc attgctggtg gccgtggccg cgttgctggt +68
        30                                            31
gggcacagcc ggccacctgt accctggaga gggtaagtct gggacaagag gcgatcacag +128
    32                  33
tatgtcgggg acacttctcc caccgcccca gccaatctag aaccctcacc ctaaggagtg +188
    34                  35
agggtcgggg acacccctct ccaccgccac agccaaccta gagaattctc cctttgggct +248

gagtaatggg gacaaccctt cccattgctc catacaactt agagcccccc aggccccacc +308
                                                          36
aaggagtcag ggttacagac acccatttca ccactccagc aatatagtcc tttcacgaga +368
                 37                                   38
gttgtggaca tttctcgttc ttcttccatc aacctagagc cccctctcca cggactgaag +428
             39   40
gttgaagact cccgtagcgt ccttgtgtct atgaagagtc ccctccctat agtgatcatc +488
```

41 42

tttacactgg ggaaggggaa gagtgggttt ggtggccctg gccctgcgtg aaacccgcgc +548

Figure S3: Genomic localization and nucleotide sequence of the Insulin Receptor gene analyzed in this work by MassArray Epityper technique. CpG dinucleotides are located in the exon and intron 1 of the Cav-1 gene (from -249 to 445 pb from ATG codon (+1)) are shadowed. ATG codon indicates the Translation Start Site (CDS). Due to the limitation of the technique, DNA methylation of some CpGs sites could not be measured (crossed out), or were measured together with others (indicated as boxed CpGs sites).
