# Supplementary material for: Expression of Caveolin 1 Is Enhanced by DNA Demethylation during Adipocyte Differentiation. Status of Insulin Signaling
Source: PLoS One. 2014 Apr 21;9(4):e95100. doi: 10.1371/journal.pone.0095100 (PMC3994010; doi:10.1371/journal.pone.0095100)
Supplement: Figure S4 — Methylation levels of the CpG dinucleotides in the insulin receptor (IR) exon and intron 1 throughout 3T3-L1 cell adipogenesis. The methylation level of 42 CpG sites in IR exon 1 and intron 1 were compared before and after 3T3-L1 adipocytic differentiation. MassARRAY system was used for the quantitative methylation analysis. Data are means ± SEM of the methylation percentage of each CpG dinucleotide specified in the figure, at days 0 and 21 of 3T3-L1 adipocyte differentiation. Groups were compared using the Mann-Whitney U test. Significant differences between day 0 and day 21 *. p≤0,05. Gene structure is schematized over the graphs, indicating the initiation codon (ATG) position. (PDF) [file pone.0095100.s004.pdf]

Insulin Receptor: Mus musculus strain C57BL/6J chromosome 8, GRCm38.p1 C57BL/6J, from 19521 bp to 20367 bp.
NCBI Reference Sequence: NC_000074.6

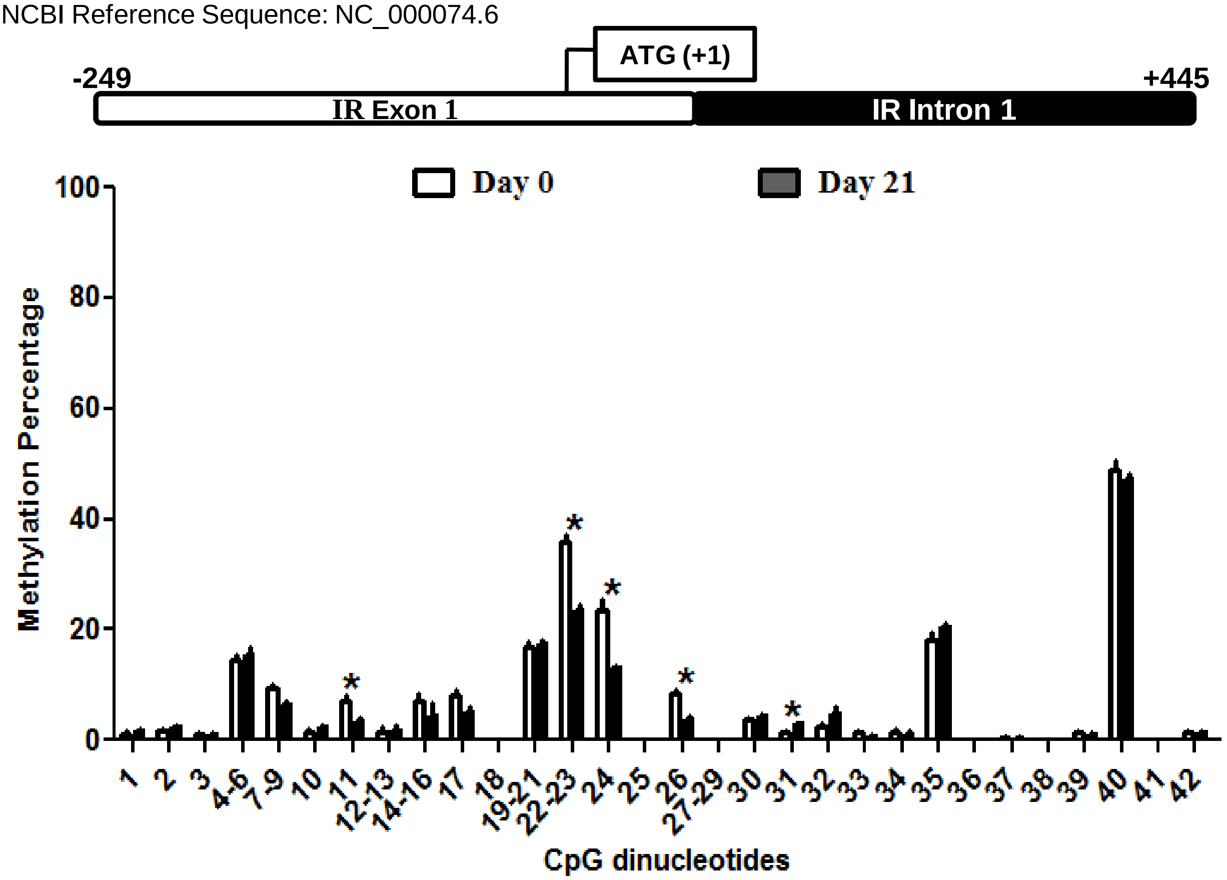

Figure S4: Methylation levels of the CpG dinucleotides in the insulin receptor (IR) exon and intron 1 throughout 3T3-L1 cell adipogenesis. The methylation level of 42 CpG sites in IR exon 1 and intron 1 were compared before and after 3T3-L1 adipocytic differentiation. MassARRAY system was used for the quantitative methylation analysis. Data are means ± SEM of the methylation percentage of each CpG dinucleotide specified in the figure, at days 0 and 21 of 3T3-L1 adipocyte differentiation. Groups were compared using the Mann-Whitney U test. Significant differences between day 0 and day 21 *. p ≤0,05. Gene structure is schematized over the graphs, indicating the initiation codon (ATG) position.
